# Supplementary material for: Choosing to view morbid information involves reward circuitry
Source: Sci Rep. 2020 Sep 17;10:15291. doi: 10.1038/s41598-020-71662-y (PMC7499173; doi:10.1038/s41598-020-71662-y)
Supplement: Supplementary file 1 — Supplementary Information. [file 41598_2020_71662_MOESM1_ESM.pdf]

Supplementary materials for:

Choosing to view morbid information involves reward circuitry.
S. Oosterwijk, L. Snoek, J. Tekoppele, L. H. Engelbert, & H. S. Scholte.

Supplementary table S1

Table S1. List of Neurosynth terms associated with the results of the exploratory whole-brain analyses for multiple contrasts. The ten terms with the highest spatial correlation with the whole-brain maps (excluding anatomical terms) are reported.

Contrast	Neurosynth term	Overall corr.	Cortical corr.	Subcortical corr.
Negative (active - passive)	Goal	.22	.24	.18
	Fear	.18	.30	-.04
	Reward	.17	.08	.43
	Task	.17	.19	.17
	Conflict	.16	.20	.02
	Anticipation	.16	.10	.40
	Monetary	.15	.04	.40
	Incentive	.14	.01	.38
	Demands	.14	.17	.01
	Incentive delay	.13	.02	.36
Positive (active - passive)	Pain	.15	.16	.28
	Painful	.14	.15	.17
	Execution	.14	.14	.07
	Somatosensory	.13	.14	.20
	Finger	.13	.12	.16
	Movement	.13	.13.	.18
	Goal	.13	.15.	.06
	Hand	.11	.11	.18
	Task	.12	.12	.19
	Tapping	.11	.12	.13
Negative (active - passive) - Positive (active - passive)	Reward	.20	.09	.48
	Task	.19	.21	.20
	Monetary	.17	.05	.44
	Semantic	.18	.22	-.02
	Anticipation	.17	.07	.43
	Incentive	.16	.02	.42
	Demands	.17	.20	.00
	Fear	.15	.22	.05
	Autobiographical	.16	.19	.02
	Retrieval	.16	.20	-.06

Supplementary table S2

Table S2. Cluster statistics and associated brain regions from the exploratory whole-brain analysis of the contrast negative $_{\text{active - passive}}$. The *X*, *Y*, and *Z* coordinates refer to MNI152 (2mm) space. The regions are taken from the Harvard-Oxford (sub)cortical atlas (Craddock et al., 2012) and voxels are assigned to regions based on their maximum probability across all ROIs within the atlas. *K* refers to the number of voxels within a particular region.

Cluster nr.	Cluster size	Cluster max.	X	Y	Z	Region	K	Max.
1	12024	6.41	34	28	-6	Left Frontal Pole	997	4.99
						Right Frontal Orbital Cortex	870	6.41
						Left Frontal Orbital Cortex	802	5.49
						Right Frontal Pole	743	5.19
						Right Inferior Frontal Gyrus, pars triang.	647	5.33
						Left Temporal Pole	640	4.64
						Left Insular Cortex	634	6.23
						Right Temporal Pole	616	4.75
						Right Insular Cortex	381	5.94
						Left Inferior Frontal Gyrus, pars operc.	373	4.53
						Left Frontal Operculum Cortex	365	5.37
						Left Inferior Frontal Gyrus, pars triang.	324	5.49
						Right Frontal Operculum Cortex	287	5.21
						Right Inferior Frontal Gyrus, pars operc.	218	4.48
						Left Middle Frontal Gyrus	192	3.80
						Right Middle Frontal Gyrus	102	3.50
						Right Middle Temporal Gyrus, anterior	39	3.63
						Left Subcallosal Cortex	39	4.23
						Left Precentral Gyrus	27	3.22
						Left Central Opercular Cortex	27	3.12
						Right Thalamus	462	4.76
						Brain-Stem, left part	447	4.19
						Left Thalamus	446	4.40
						Left Caudate	257	4.39
						Right Caudate	239	4.95
						Right Putamen	211	4.53
						Left Putamen	173	4.88
						Brain-Stem, right part	153	4.34
						Left Accumbens	61	4.16
						Right Pallidum	34	3.63
						Right Accumbens	29	4.31
						Right Amygdala	26	3.69
2	7133	6.17	8	30	26	Right Paracingulate Gyrus	974	5.95
						Right Cingulate Gyrus, anterior division	897	6.17
						Left Paracingulate Gyrus	835	5.99

						Left Superior Frontal Gyrus	832	4.73
						Left Cingulate Gyrus, anterior division	797	5.89
						Right Superior Frontal Gyrus	646	4.67
						Right Frontal Pole	443	4.25
						Left Juxtapositional Lobule Cortex	360	5.04
						Left Postcentral Gyrus	287	4.15
						Left Precentral Gyrus	283	4.08
						Right Juxtapositional Lobule Cortex	281	4.78
						Left Frontal Pole	179	4.11
						Left Middle Frontal Gyrus	124	4.03
						Left Superior Parietal Lobule	42	3.64
						Right Cingulate Gyrus, posterior division	40	3.41
						Right Frontal Medial Cortex	23	3.13
3	534	5.05	-34	-46	-24	Left Temporal Fusiform Cortex, posterior	95	3.98
						Left Temporal Occipital Fusiform Cortex	76	5.05
4	401	4.13	20	-50	0	Right Lingual Gyrus	182	4.13
						Right Intracalcarine Cortex	119	3.77
						Right Supracalcarine Cortex	38	3.47
						Right Cingulate Gyrus, posterior division	35	3.78
5	375	4.70	-16	-50	-2	Left Lingual Gyrus	115	4.70
						Left Cingulate Gyrus, posterior division	111	4.05
						Left Precuneous Cortex	61	3.97
						Left Intracalcarine Cortex	43	3.05

Supplementary table S3

Table S3. Cluster statistics and associated brain regions from the exploratory whole-brain analysis of the contrast positive $_{\text{active - passive}}$. The *X*, *Y*, and *Z* coordinates refer to MNI152 (2mm) space. The regions are taken from the Harvard-Oxford (sub)cortical atlas (Craddock et al., 2012) and voxels are assigned to regions based on their maximum probability across all ROIs within the atlas. *K* refers to the number of voxels within a particular region.

Cluster nr.	Cluster size	Cluster max.	X	Y	Z	Region	K	Max.
1	1857	4.40	0	6	48	Right Cingulate Gyrus, anterior division	400	4.10
						Left Juxtapositional Lobule Cortex	347	4.40
						Left Cingulate Gyrus, anterior division	347	4.34
						Right Paracingulate Gyrus	208	4.02
						Right Juxtapositional Lobule Cortex	164	4.27
						Left Paracingulate Gyrus	158	4.27
						Right Cingulate Gyrus, posterior division	67	3.55
						Left Superior Frontal Gyrus	51	4.06
						Left Precentral Gyrus	40	3.35

						Left Cingulate Gyrus, posterior division	28	3.22
2	1651	4.54	50	36	2	Right Frontal Pole	463	4.54
						Right Inferior Frontal Gyrus, pars triang.	304	4.51
						Right Frontal Orbital Cortex	240	3.95
						Right Insular Cortex	228	4.21
						Right Temporal Pole	143	3.66
						Right Frontal Operculum Cortex	117	4.12
						Right Superior Temporal Gyrus, anterior	48	3.44
						Right Superior Temporal Gyrus, posterior	36	3.19
						Right Inferior Frontal Gyrus, pars operc.	27	3.02
3	1196	4.57	-40	14	-8	Left Insular Cortex	421	4.57
						Left Frontal Pole	377	4.51
						Left Frontal Orbital Cortex	128	3.70
						Left Frontal Operculum Cortex	107	3.82
						Left Inferior Frontal Gyrus, pars triang.	59	3.67
						Left Putamen	40	3.55
4	931	4.84	-34	-46	-20	Left Temporal Occipital Fusiform Cortex	60	4.84
						Left Temporal Fusiform Cortex, posterior	44	4.11
5	570	3.98	-48	-26	42	Left Postcentral Gyrus	300	3.98
						Left Precentral Gyrus	220	3.83
						Left Supramarginal Gyrus, anterior	35	3.27
6	422	3.95	-26	-26	64	Left Postcentral Gyrus	136	3.43
						Left Lateral Occipital Cortex, superior	117	3.85
						Left Superior Parietal Lobule	82	3.65
						Left Precentral Gyrus	67	3.95
7	332	4.11	26	-68	-4	Right Lingual Gyrus	149	3.62
						Right Intracalcarine Cortex	97	3.33
						Right Supracalcarine Cortex	39	3.75
						Right Occipital Fusiform Gyrus	25	4.11
8	266	3.84	-8	-18	4	Right Thalamus	128	3.71
						Left Thalamus	127	3.84

Supplementary Table S4

Table S4. Stimulus codes for the images used in the choice task. Images were taken from both the IAPS (Lang, Bradley, & Cuthbert, 2008) and NAPS (Marchewka, Żurawski, Jednoróg, & Grabowska, 2014) database.

**Image valence**	**Image selected from following database:**	
	IAPS database	*NAPS database*
Negative images	IAPS 2683	NAPS faces 16
	IAPS 2691	NAPS faces 28
	IAPS 2799	NAPS faces 283
	IAPS 3216	NAPS faces 7
	IAPS 6212	NAPS people 127
	IAPS 6313	NAPS people 201
	IAPS 6520	NAPS people 214
	IAPS 6571	NAPS people 22
	IAPS 6831	NAPS people 226
	IAPS 6840	NAPS people 3
	IAPS 9050	NAPS people 33
	IAPS 9163	NAPS people 39
	IAPS 9250	
	IAPS 9400	
	IAPS 9419	
	IAPS 9427	
	IAPS 9428	
	IAPS 9429	
	IAPS 9433	
	IAPS 9435	
	IAPS 9900	
	IAPS 9921	
	IAPS 9926	
Positive images	IAPS 2091	NAPS faces 107
	IAPS 2216	NAPS faces 109
	IAPS 2299	NAPS faces 232
	IAPS 2332	NAPS faces 234
	IAPS 2340	NAPS faces 3
	IAPS 4623	NAPS faces 321
	IAPS 7515	NAPS faces 354
	IAPS 7660	NAPS faces 61
	IAPS 8185	NAPS faces 82
	IAPS 8461	NAPS faces 88
	IAPS 8496	NAPS faces 89
	IAPS 8540	NAPS people 168
		NAPS people 174
		NAPS people 181
		NAPS people 182
		NAPS people 185
		NAPS people 186
		NAPS people 187
		NAPS people 192
		NAPS people 228
		NAPS people 43
		NAPS people 50
		NAPS people 54

Supplementary Figure S1

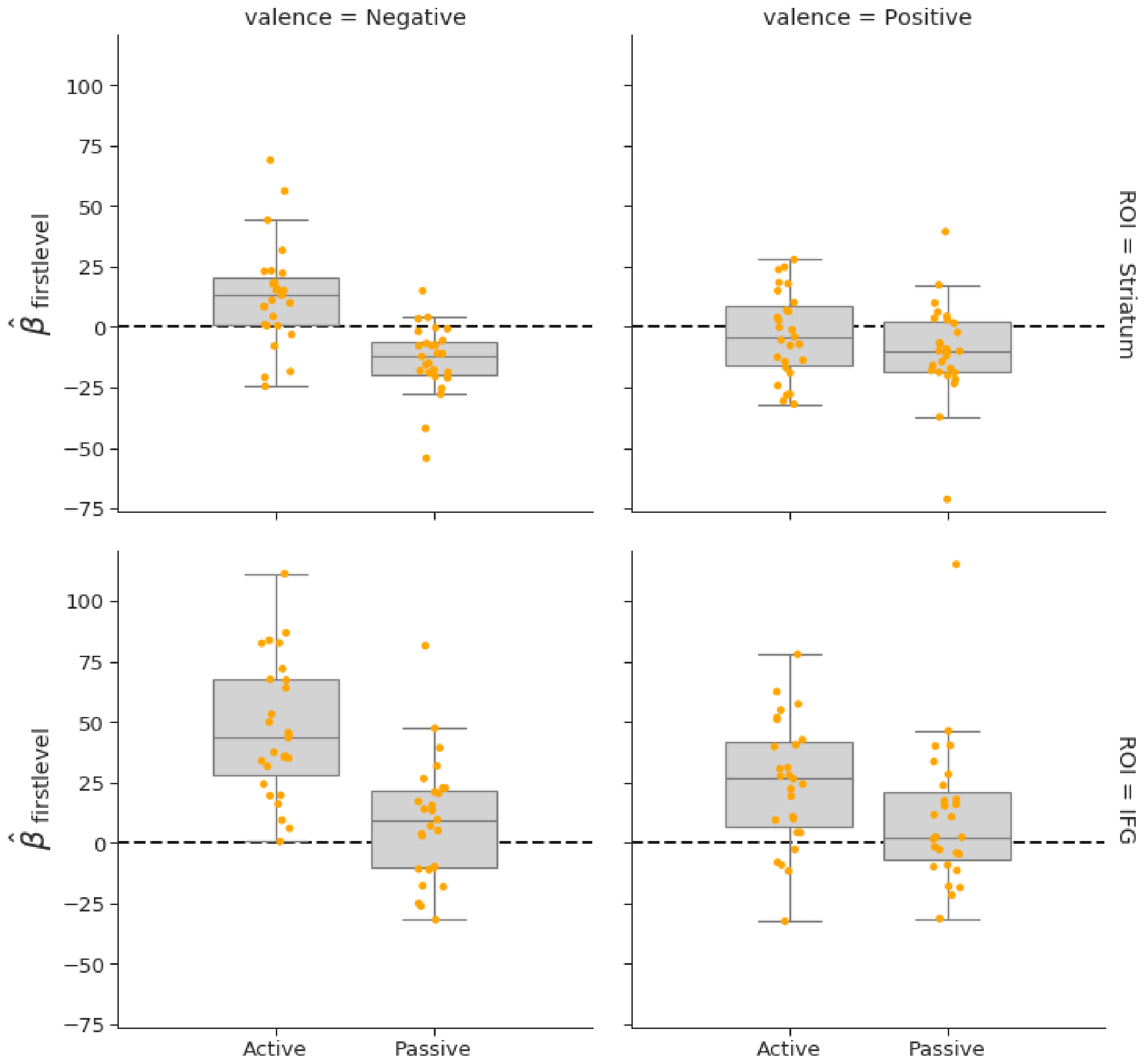

Figure S1. Subplots of individual regressors from the significant voxels in the confirmatory contrast negative $_{\text{active - passive}}$ - positive $_{\text{active - passive}}$ in the induction-phase. These plots show the direction of the effects. Plots are averaged over all significant voxels within each ROI (striatum in upper plots, IFG in lower plots), separately for the negative trials (left plots) and positive trials (right plots) with subplots for the active choice and passive viewing condition. Dots represent the participant-specific ROI-average parameter estimate from the first-level analysis. The horizontal line in the boxplots represents the median and the whiskers represent the interquartile range. Note that this figure is only meant to show the directionality of the effects, not their statistical significance (as the ROIs itself only contain voxels that were significant in the group-analysis).
